# Bilateral adrenal uptake of ^123^I MIBG scintigraphy with mild catecholamine elevation, the diagnostic dilemma, and its characteristics

**DOI:** 10.1038/s41598-022-13132-1

**Published:** 2022-06-03

**Authors:** Yuiko Inaba, Masaaki Yamamoto, Shin Urai, Masaki Suzuki, Seiji Nishikage, Maki Kanzawa, Yayoi Aoyama, Tomonori Kanda, Katsumi Shigemura, Hironori Bando, Genzo Iguchi, Yasuhiro Nakamura, Masato Fujisawa, Akihisa Imagawa, Hidenori Fukuoka, Wataru Ogawa

**Affiliations:** 1 Division of Diabetes and Endocrinology, Department of Internal Medicine, Kobe University Hospital, 7-5-2 Kusunoki-cho, Chuo-ku, Kobe, 650-0017 Japan; 2 Department of Internal Medicine(I), Osaka Medical and Pharmaceutical University, 2-7 Daigakumachi, Takatsuki, Osaka 569-8686 Japan; 3 Division of Diabetes and Endocrinology, Kobe University Graduate School of Medicine, 7-5-1 Kusunoki-cho, Chuo-ku, Kobe, 650-0017 Japan; 4 Department of Diagnostic Pathology, Kobe University Hospital, 7-5-2 Kusunoki-cho, Chuo-ku, Kobe, 650-0017 Japan; 5 Department of Pathology, Tohoku University Hospital, 1-1 Seiryo-machi, Aoba-ku, Sendai, Miyagi 980-8574 Japan; 6 Department of Radiology, Kobe University Graduate School of Medicine, 7-5-1 Kusunoki-cho, Chuo-ku, Kobe, 650-0017 Japan; 7 Division of Urology, Department of Organ Therapeutics, Faculty of Medicine, Kobe University Graduate School of Medicine, 7-5-1 Kusunoki-cho, Chuo-ku, Kobe, 650-0017 Japan; 8 Department of Public Health, Kobe University Graduate School of Health Science, 7-5-1 Kusunoki-cho, Chuo-ku, Kobe, 650-0017 Japan; 9 Division of Development of Advanced Therapy for Metabolic Disease, Kobe University Graduate School of Medicine, 7-5-1 Kusunoki-cho, Chuo-ku, Kobe, 650-0017 Japan; 10 Medical Center for Student Health, Kobe University, 1-1, Rokkodai-cho, Nada-ku, Kobe, 657-8501 Japan; 11 Department of Biosignal Pathophysiology, Kobe University Graduate School of Medicine, 7-5-1 Kusunoki-cho, Chuo-ku, Kobe, Hyogo 650-0017 Japan; 12 Division of Pathology, Tohoku Medical and Pharmaceutical University, 4-4-1 Komatsushima, Aobaku, Sendai, Miyagi 981-8558 Japan

**Keywords:** Endocrinology, Endocrine system and metabolic diseases, Endocrine cancer

## Abstract

Cases in which bilateral adrenal ^123^I-Metaiodobenzylguanidine (^123^I-MIBG) scintigraphy accumulation is sometimes shown, with mildly elevated catecholamine (CA) or metanephrine (MN) levels (within 3 times the upper reference limit) are diagnostic dilemmas. We experienced 3 cases of adrenal incidentalomas with this dilemma in the differential diagnosis. The clinical diagnosis was subclinical Cushing's syndrome in 2 cases, and primary aldosteronism in 1. Despite suspected CA excess in clinical symptoms and imaging findings, the pathological findings of all these tumors were revealed to be cytochrome P450 family 11 subfamily B member 1 (CYP11B1) positive adrenocortical adenomas. Interestingly, adrenal medullary hyperplasia (AMH) was detected in the adrenal parenchyma of all those backgrounds. To clarify the clinical features of such cases, a cross-sectional study was conducted at the Kobe University Hospital from 2014 to 2020. One-hundred sixty-four patients who had undergone ^123^I-MIBG scintigraphy were recruited. Among them, 10 patients (6.1%) met the above criteria, including the presented 3 cases. Plasma adrenaline, noradrenaline, urinary metanephrine, and normetanephrine had values of 0.05 ± 0.05 ng/mL, 0.63 ± 0.32 ng/mL, 0.22 ± 0.05 mg/day, and 0.35 ± 0.16 mg/day, respectively. Nine cases were complicated with hypertension, and symptoms related to CA excess were observed. Half of them (5 cases) including presented 3 cases had unilateral adrenal tumors. These suggest that in cases of bilateral adrenal uptake on ^123^I-MIBG, AMH needs to be considered. Adrenocortical adenomas may be associated with AMH and further larger investigation is needed for this pathology.

## Introduction

^123^I-MIBG scintigraphy, a noradrenaline analog that is taken up into cells via the noradrenaline transporter^1^, is a highly functional imaging tool used for the diagnosis of pheochromocytoma and paraganglioma (PPGL)^2^. Tracer accumulation in scintigraphy has a high sensitivity of 82–88% and a high specificity of 82–84% for detecting PPGL^3^. While this image inspection is highly accurate, it should be noted that there are several false-negative tumors, such as malignant PPGL^3^, paraganglioma, multiple tumors, and hereditary PPGL, including germline succinate dehydrogenase (*SDHx*) mutations^4^. Although only a few cases have false positives in ^123^I-MIBG^5,6^, there are still cases in which the diagnosis of PPGL is uncertain, such as the bilateral adrenal parenchyma uptake on ^123^I-MIBG scintigraphy, with mild elevation of CA or MN levels.

In adrenal incidentalomas, it is necessary to accurately rule out pheochromocytomas because of its perioperative risk. The combination of resting plasma CAs ≧ 2000 pg/mL and urinary MNs ≧ 1.8 mg/24 h, that is, more than 3 times the upper reference limit of each, has a diagnostic accuracy of 98% in pheochromocytoma^7^. Therefore, especially, in cases of bilateral adrenal accumulation of ^123^I-MIBG and mild elevation of CA levels are diagnostic dilemmas. Because, in the case of pheochromocytomas, premedication is needed for surgery. Controlling blood pressure, heart rate, and arrhythmia and correcting circulating plasma volume with preoperative administration of α-receptor blockers and metirosine are known to reduce perioperative risk in patients with pheochromocytomas^8,9^. It should also be noted that bilateral pheochromocytoma has a higher frequency of being heritable and malignant than unilateral pheochromocytoma^10–12^. Moreover, the case with bilateral accumulation of ^123^I-MIBG includes AMH, a precursor lesion for pheochromocytoma, which is frequently observed in multiple endocrine neoplasia type 2 (MEN2)^13^. In these cases, ^123^I-MIBG positive adrenal glands may be detected even before the tumor is observed^13^. For these reasons, it is clinically quite important to conduct a detailed investigation of such cases with diagnostic dilemma for which pheochromocytoma cannot be ruled out. In this paper, we presented three adrenal incidentaloma cases that showed bilateral adrenal uptake of ^123^I-MIBG with mild CA excess and were performed adrenalectomy. We further extracted cases matching the above conditions from cases in which ^123^I-MIBG scintigraphy had been performed at our institute and analyzed their clinical features.

## Methods

### Hormone measurement

Plasma CA levels were measured in the morning after overnight fasting in the supine position. For the collection of 24-h urinary fractionated MNs and CAs, all participants were instructed to abstain from caffeinated foods and drinks for at least 48 h. Plasma CA levels were measured by high-performance liquid chromatography (HPLC) (LSI Medience Corporation, Tokyo, Japan), enzyme immunoassay (BML, Inc., Tokyo, Japan), and radioimmunoassay (LSI Medience Corporation, Tokyo, Japan), respectively. The intra- and inter-assay coefficients of variation for each hormone assay were as follows: plasma adrenaline (Ad), < 4.08% and < 2.23%; noradrenaline (NA), < 9.34% and < 2.27%; and dopamine (DA), < 8.96% and < 2.89%. Urinary MN and CA levels were measured by HPLC (LSI Medience Corporation, Tokyo, Japan). The intra- and inter-assay coefficients of variation for each hormone assay were as follows: urinary Ad, < 6.21% and < 6.35%; urinary NA, < 4.09% and < 3.82%; urinary DA, < 5.32% and < 4.46%; urinary MN, < 1.4% and < 6.2%; and urinary normetanephrine (NMN), < 0.7% and < 5.3%. Serum cortisol levels were measured by an enzyme immunoassay (Electro Chemiluminescence Immunoassay and Enzyme Immunoassay; TOSOH, Tokyo, Japan). Plasma aldosterone concentrations (PAC) were measured by radioimmunoassay (RIA) (SPAC-S Aldosterone Kits, Fuji Rebio, Co., Ltd, Tokyo, Japan).

### Pathological and immunohistochemical analysis

We performed detailed pathological analysis in 3 patients (Cases 1 to 3) who had undergone adrenalectomy after ^123^I-MIBG scintigraphy. The histological definition was based on the World Health Organization (WHO) expert consensus proposal in 2017^14^. In this proposal, AMH was defined when the medulla was more than a third of the adrenal thickness, in the absence of cortical atrophy, and/or when the medulla was noted in the tail of the gland. We further examined the expression of adrenocortical steroidogenic enzymes associated with cortisol and aldosterone, including CYP11B1 and CYP subfamily B member 2 (CYP11B2), in these tumors and non-tumor components of the excised specimens. For immunohistochemistry, we used anti-CYP11B1 antibody (rat, monoclonal) and anti-CYP11B2 antibody (mouse, monoclonal), both of which were developed in the laboratory of Dr. Gomez Sanchez (University of Mississippi Medical Center, Jackson, MS)^15^. We further examined using the specimens of the typical functioning adrenocortical adenoma cases. We evaluated the presence or absence of AMH in the background adrenal parenchyma in cases clinically diagnosed with Cushing's syndrome (n = 5) and cases diagnosed with primary aldosteronism (n = 5). For the analysis of signal transducer and activator of transcription 3 (STAT3) phosphorylation status in these specimens, we used phospho-serine (p-S) 727 STAT3 (1:200, Cell Signaling Technology), and phospho-tyrosine (p-Y) 705 STAT3 (1:200, Cell Signaling Technology) antibodies as previously described^16^.

### ^123^I-MIBG scintigraphy planar protocol

Patients received thyroid blockade with a saturated solution of potassium iodide before ^123^I-MIBG scintigraphy (50 mg/day starting 4 days before tracer injection). Whole-body planar and tomographic (single photon emission computed tomography (SPECT)) images were obtained 24 h after intravenous administration of 111 MBq ^123^I-MIBG. An OptimaNM/CT640 dual-head gamma camera (GE Healthcare) was used, and SPECT was combined with low-dose computed tomography (CT) for anatomic co-registration. The SPECT scan was acquired with energy windows for I^123^ centered at 159.0 keV (photo peak window), a 64 × 64 matrix of 8.8 mm pixel size, and a total of 60 projections (30 steps) over 360° with a dwell time of 45 s/step. SPECT images were reconstructed using three-dimensional ordered subset expectation maximization (3D-OSEM) with 2 iterations, 10 subsets, and a Butterworth filter.

### Patients and design

This is a retrospective cross-sectional study of consecutive patients who had undergone ^123^I-MIBG scintigraphy because of suspected PPGL at the Kobe University Hospital from April 2014 to September 2020. We included a total of 164 patients who had undergone whole-body planar and selected SPECT 24 h after the administration of ^123^I-MIBG during the study period (Fig. 4). These patients were suspected to have PPGL due to adrenal tumors and/or the presence of symptoms associated with pheochromocytomas showing CA or MN excess findings. Their need for ^123^I-MIBG was determined by each attending physician including other than endocrinologist. We excluded patients who had already undergone adrenalectomy at the time of ^123^I-MIBG scintigraphy. Next, we selected 17 patients with bilateral adrenal uptake on ^123^I-MIBG scintigraphy. The uptake of ^123^I-MIBG in the adrenal glands was semi-quantified compared to the hepatic uptake. We calculated the adrenal-to-liver (A/L) ratios as the mean counts per pixel in the segmented adrenal volume of interest over the mean counts per pixel in the liver volume of interest. These cases were visually delineated on planar images, and a region of interest (ROI) was manually drawn around the adrenal gland. A circular ROI of 5 cm in diameter was positioned centrally in the right upper lobe of the liver, and A/L ratios were calculated. We defined positive scintigraphic uptake as more intense in the adrenal glands than in the liver^17^. Then, we selected patients either whose one of the following was elevated within 3 times the upper reference limit: plasma Ad, plasma NA, urinary MN, or urinary NMN measurements, and extracted 10 patients. These are cases that do not meet the diagnostic criteria for pheochromocytoma^2^. We confirmed that these patients were not taking any medications that have shown to affect plasma CA or urinary MN levels in the clinical practice guideline for diagnosis^18^. We analyzed the background characteristics of them including sex, age, CA and MN measurements, and glycosylated hemoglobin (HbA1c) levels. We also assessed their clinical features, including the presence of hypertension, subjective symptoms associated with excess CA, and adrenal tumors. Abdominal CT (non-contrast) had been performed in all 10 cases. In cases with adrenal tumors, we also evaluated tumor sizes and CT values. All processes were conducted in compliance with the protocol reviewed and approved and need of the informed consent was waived by the Ethics Committee of Kobe University Graduate School of Medicine (Permit Number: #1351). Written informed consent for participation was not required for this study in accordance with the national legislation and the institutional requirements. All methods were carried out in accordance with relevant guidelines and regulations.

### Genetic analysis of FGFR4

Recently, we identified *fibroblast growth factor receptor 4 (FGFR4)* variant (G388R) as potential associated pathogenesis between adrenocortical adenomas and CA excess from exome analysis (PMID: 32803097). Therefore, we focused on whether this variant is associated with the pathogenesis between AMH and adrenocortical adenomas. According to the manufacturer's instructions, genomic DNA was isolated from formalin-fixed paraffin-embedded of adrenal tumor samples using a genomic DNA extraction kit (QIAGEN Inc., Hilden, Germany). To perform gene analysis of the *FGFR4*-G388R variant, coding sequences of the gene were amplified by polymerase chain reaction and Sanger sequencing was performed. The primers used for the analysis were as follows: *forward*, 5′-GGCCAGGTATACGGACATCATCC-3′; *reverse*: 5′-AGAGGGAAGCGGGAGAGCTTCTG-3′.

## Results

### Three cases presentation

First, we present three adrenal incidentaloma cases who showed mild CA excess with showing bilateral adrenal uptake of ^123^I-MIBG but underwent unilateral adrenalectomy.

#### Case 1

59 years old male. He was found a 16 mm-sized right adrenal tumor with a low CT value (− 5 Hounsfield units (HU)). He had hypertension, palpitation, and weight loss. He was diagnosed with primary aldosteronism due to impaired PAC suppression during the saline infusion test (PAC 80.0 pg/mL). Plasma adrenocorticotropic hormone (ACTH) levels were 16.6 pg/mL and serum cortisol levels (F) after overnight 1 mg-dexamethasone suppression test (DST) was 1.0 μg/dL. No cortisol hypersecretion was observed. However, his daily urinary MN levels showed mild elevation (0.31 mg/day).

#### Case 2

46 years old female. She had a 42 mm-sized left adrenal tumor with a CT value of 13 HU. She had hypertension, palpitation, and headache. Regarding hormonal functionality, Plasma ACTH levels was 1.00 pg/mL. She was diagnosed with subclinical Cushing's syndrome due to impaired suppression F of after 1 mg-DST (F 11.2 μg/dL). ^131^I-adosterol scintigraphy showed uptake into the tumor while suppressing the contralateral adrenal gland. However, her plasma NA levels (0.97 ng/mL), and daily urinary MN levels (0.22 mg/day) showed mild elevation.

#### Case 3

47 years old male. He had a 36 mm-sized left adrenal tumor, and the CT values showed high (45 HU). He had hypertension, palpitation, and weight loss. Plasma ACTH levels was 2.2 pg/mL. He was diagnosed with subclinical Cushing's syndrome due to impaired suppression of F after 1 mg-DST (F 16.87 μg/dL). His ^131^I-adosterol scintigraphy showed uptake into the tumor while suppressing the contralateral adrenal gland, as shown in Case 2. However, both plasma NA levels (0.62 ng/mL) and daily urinary NMN levels (0.29 mg/day).

**Figure 1:**
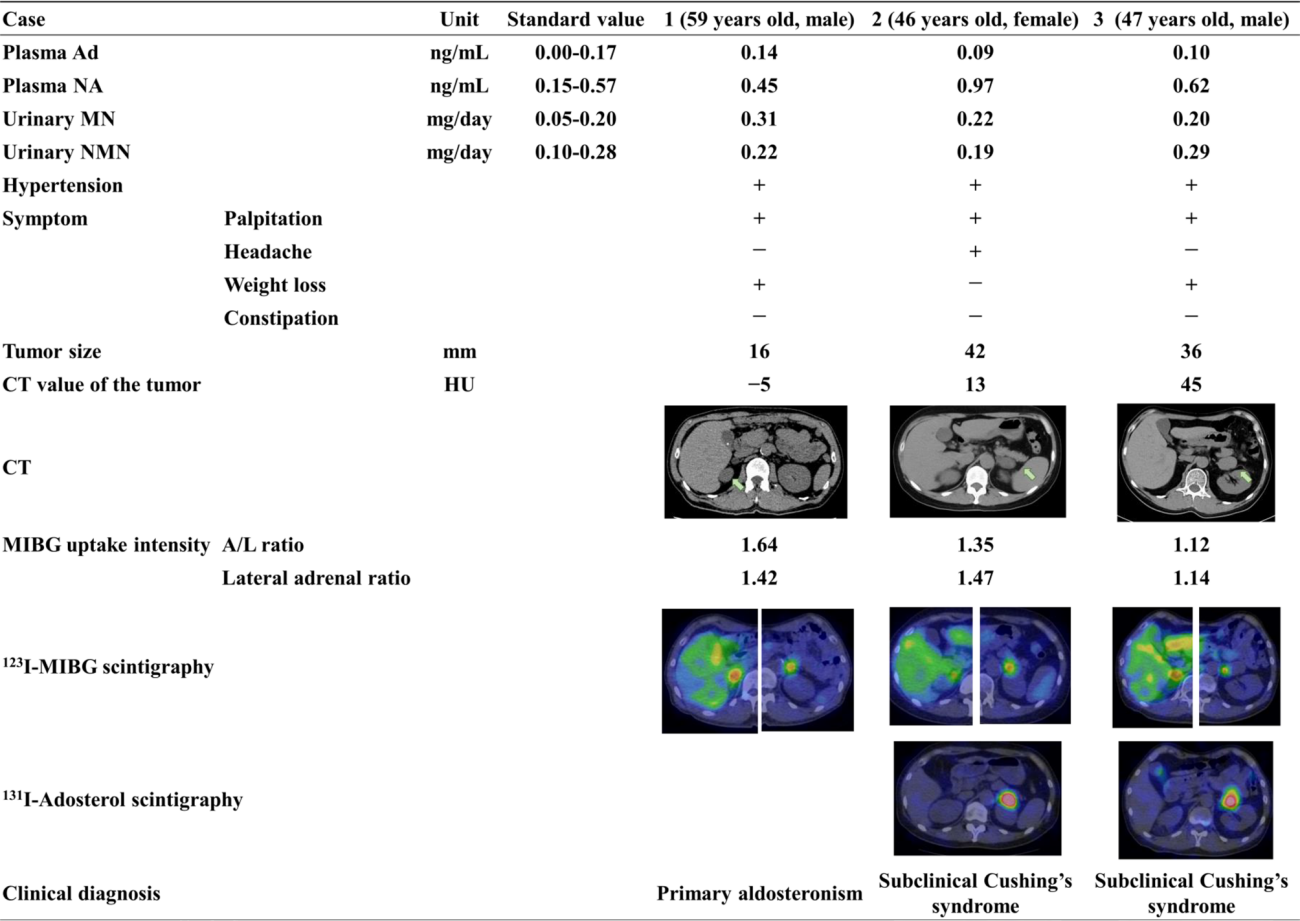
^123^I-MIBG scintigraphy showed uptake in the bilateral adrenal glands in 3 cases.

Since the possibility of pheochromocytoma could not be clinically ruled out due to symptoms and hormonal levels in all cases, ^123^I-MIBG scintigraphy was performed and showed uptake in the bilateral adrenal glands. Cases 1 and 2 showed strong ^123^I-MIBG uptake on the ipsilateral side of the tumor. Conversely, Case 3 rather showed predominant ^123^I-MIBG accumulation in the adrenal gland on the opposite side of the tumor. In addition, we evaluated whether the ^123^I-MIBG accumulation site on the tumor-side was in a tumor or a normal adrenal gland. In Case 1, ^123^I-MIBG accumulation seemed to have accumulated in the tumor. In Case 2 and Case 3, unlike the accumulation site of ^131^I-adosterol scintigraphy, ^123^I-MIBG accumulation appeared in the normal gland (Fig. 1).

### Pathological analysis

**Figure 2:**
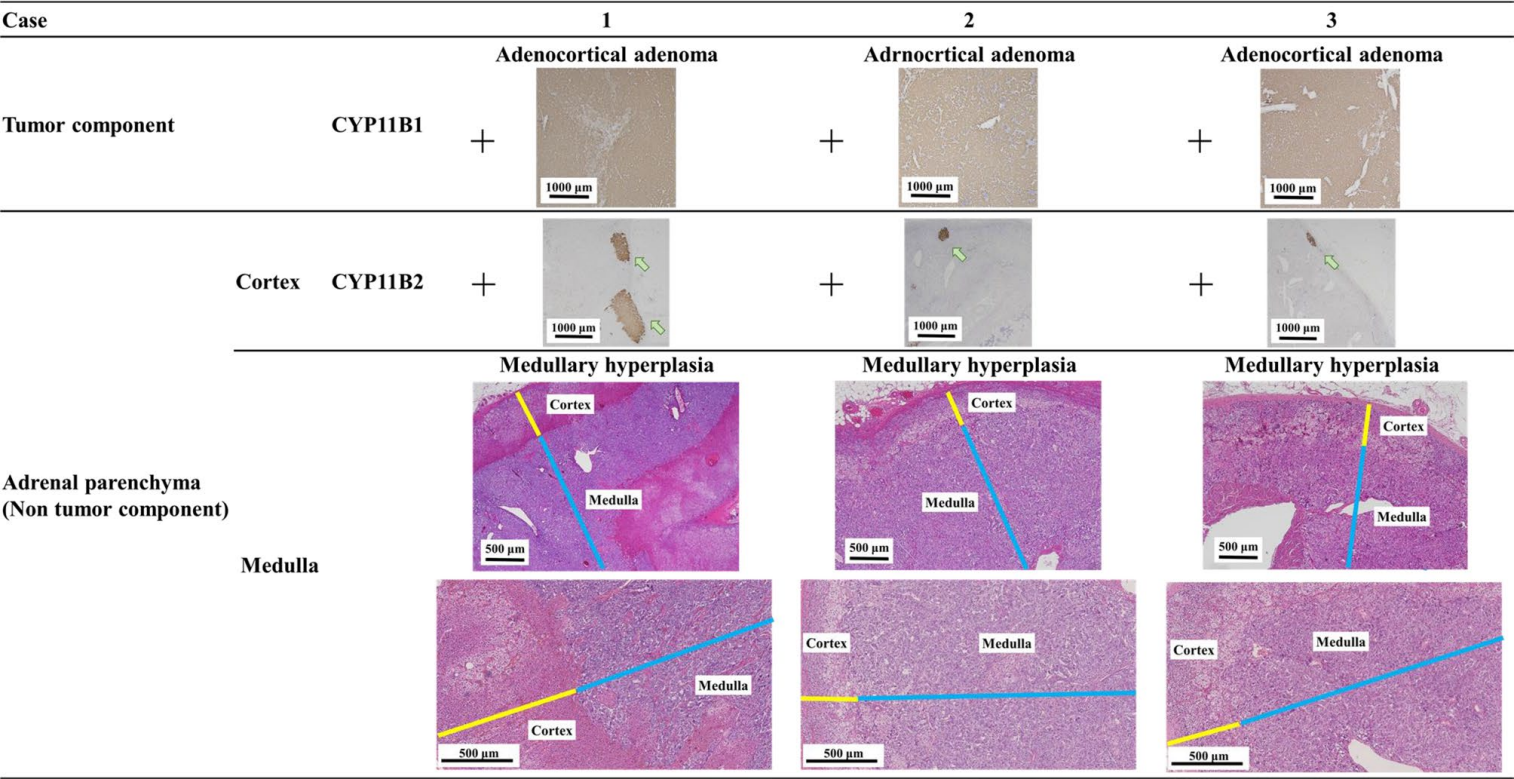
All the adrenal tumors were cortical adenomas and CYP11B1 positive images were observed. CYP11B2 were locally positive in the non-tumor part (arrows). Background adrenal parenchyma showed adrenal medullary hyperplasia in all 3 cases.

**Figure 3:**
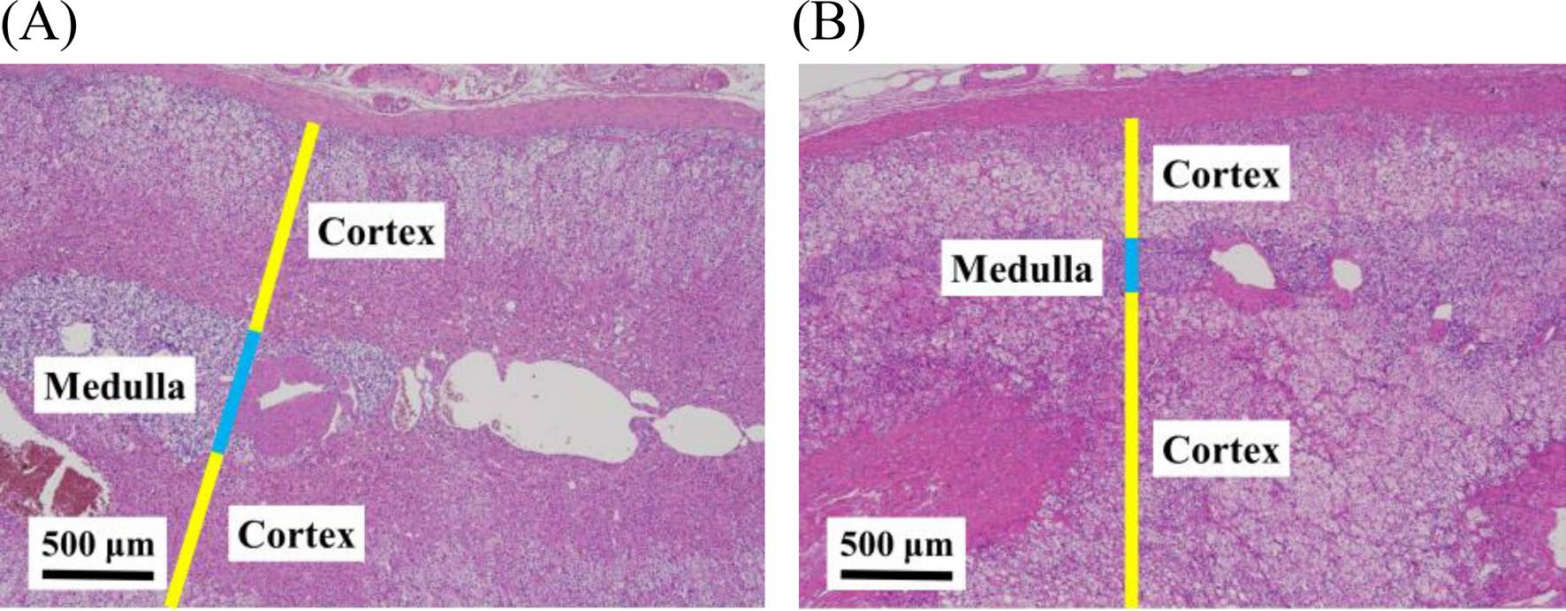
Adrenal parenchyma behind typical functional adenocortical adenomas. (**A**) Adrenal parenchyma behind a typical cortisol-producing adenoma (**B**) Adrenal parenchyma behind a typical aldosterone-producing adenoma.

Next, these resected tumors and associated adrenal glands were examined pathologically (Fig. 2). Interestingly, in the adrenal glands of all these 3 specimens, medulla thickness was greater than one-third of the entire adrenal gland, or medulla tissue was observed from the entire adrenal gland including the tail. In these specimens, adrenocortical atrophy was not observed, indicating the diagnosis of AMH. Despite the uptake of ^123^I-MIBG, besides, all the tumor components were adrenocortical adenomas. Further immunostaining with adrenocortical enzymes revealed that all these tumor components were CYP11B1 positive and CYP11B2 negative, indicating cortisol-producing tumors. In contrast, focal CYP11B2 positive clusters were found within the associated adrenal glands but not in the tumors, indicating the presence of multiple aldosterone producing micronodule (Fig. 2). To investigate whether this AMH change is seen in the background adrenal glands of typical functioning adrenocortical adenomas, we randomly selected specimens of Cushing’s syndrome cases (n = 5), and primary aldosteronism cases (n = 5) and evaluated pathologically. No evidence of AMH was found in the tumor-side background adrenal parenchyma of typical adrenal Cushing's syndrome and primary aldosteronism (Fig. 3).

### Case detection who showed ^123^I-MIBG bilateral adrenal accumulation and mild CA elevation

**Figure 4:**
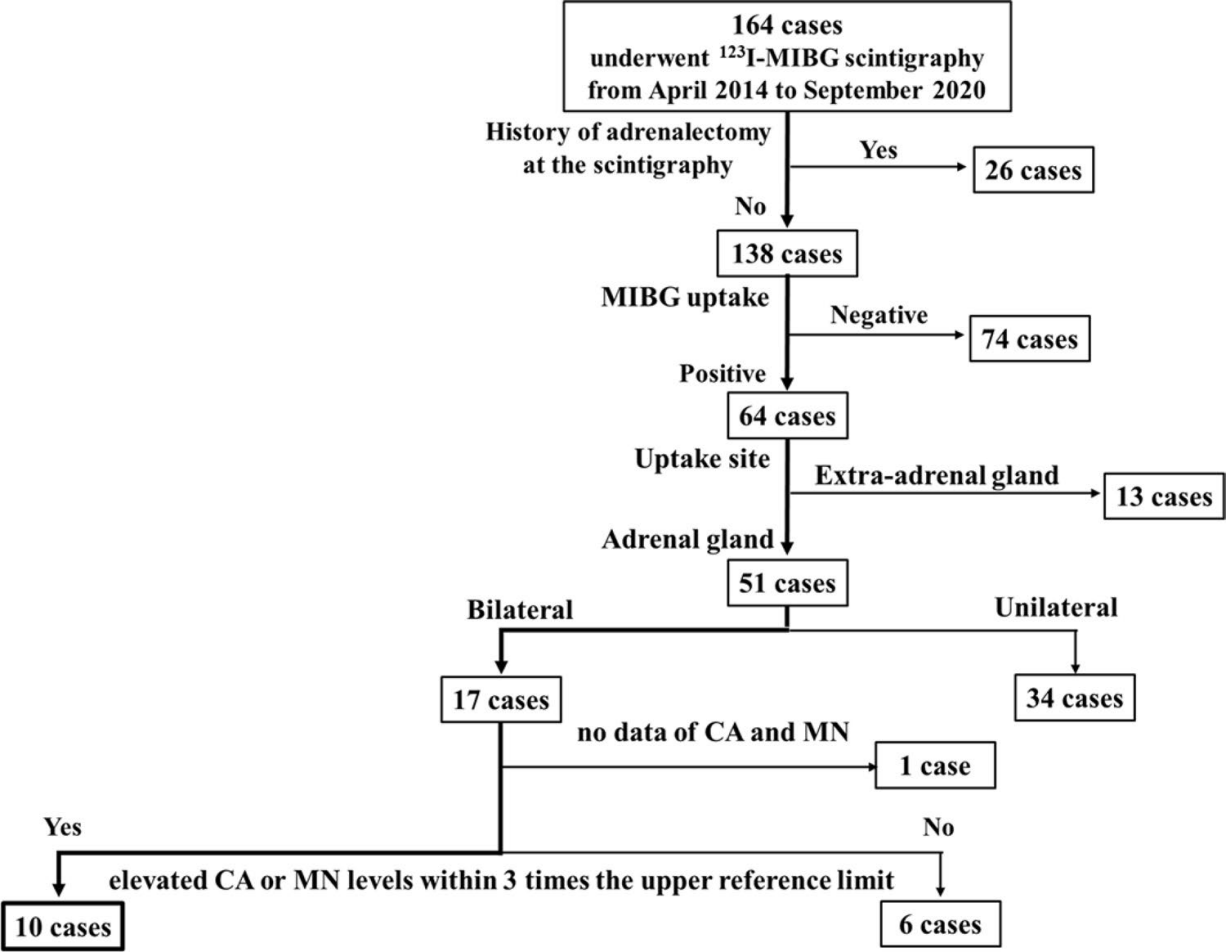
Catecholamine (CA); plasma adrenaline (Ad) and noradrenaline (NA) levels, metanephline (MN); urinary fractionated MN and normetanephrine (NMN).

Of the 164 patients who underwent ^123^I-MIBG scintigraphy at our hospital, 158 had undergone both whole body planar and SPECT image, and 26 were excluded because of a previous adrenalectomy. Of these, 64 cases showed positive accumulation, and 51 cases showed ^123^I-MIBG accumulation in the adrenal gland. Furthermore, 17 patients of them showed bilateral accumulation of the gland. Among these bilateral adrenal accumulations of ^123^I-MIBG, we extracted 10 cases in which either plasma CA or urinary MN levels were above the upper limit of the reference values and was less than 3 times the upper limit, specifically 0.17 < plasma Ad < 0.51 ng/mL, 0.57 < plasma NA < 1.71 ng/mL, 0.20 < urinary MN < 0.60 mg/day, or 0.28 < urinary NMN < 0.84 mg/day (Fig. 4). These 10 cases include the presented 3 cases above (Cases 1 to 3).

Of the 10 patients, 8 were males and 2 were females, with a median age of 53 years (Table 1). Patients with the above criteria tended to be more common in males. The plasma NA levels were slightly elevated and were less than 3 times the upper limit (0.57 [0.46–0.89] ng/mL), while plasma Ad levels were within a normal range (0.04 [0.02–0.09] ng/mL). In the meantime, both daily urinary MN and NMN levels showed mild elevation (0.22 [0.20–0.23] and 0.33 [0.23–0.38] mg/day), respectively. Nine patients had hypertension with the following symptoms suspected to be CA excess, including palpitation (50%), headache (30%), weight loss (20%), and constipation (20%). The A/L ratio and the lateral adrenal ratio of ^123^I-MIBG uptake intensities were 1.29 [1.13–1.67] and 1.22 [1.09–1.39], respectively. Among these 10 cases, 5 had adrenal tumors, and 3 of them (Cases 1 to 3) had undergone tumor-side adrenalectomy. There was no apparent difference in the degree and symptoms of excess CA between these 3 cases and the other 7 patients who had not undergone surgery. None of these 10 patients had a family history of hereditary pheochromocytoma, including von Hippel-Lindau, neurofibromatosis type1 (NF1), and MEN 2. None of the present 10 cases had complications related to Carney complex.

**Table 1:** Clinical characteristics of 10 cases with bilateral adrenal accumulation with MIBG and a mild increase in CA or MN.

Case	Unit	Standard value	1	2	3	4	5	6	7	8	9	10	Median [range]
Sex			Male	Female	Male	Male	Male	Female	Male	Male	Male	Male	
Age	Years		59	46	47	66	41	64	72	47	83	37	53 [46–66]
Plasma Ad	ng/mL	0.00–0.17	0.14	0.09	0.10	0.00	0.02	0.09	0.02	0.04	0.04	0.00	0.04 [0.02–0.09]
Plasma NA	ng/mL	0.15–0.57	0.45	0.97	0.62	0.58	0.02	0.55	1.00	0.04	1.20	0.00	0.57 [0.46–0.89]
Urinary MN	mg/day	0.05–0.20	0.31	0.22	0.20		0.19	0.26	0.22	0.22		0.12	0.22 [0.20–0.23]
Urinary NMN	mg/day	0.10–0.28	0.22	0.19	0.29		0.67	0.23	0.42	0.37		0.37	0.33 [0.23–0.38]
HbA1c	%		5.7	5.6	5.7	5.9	6.0	8.1	5.2	5.7	6.0	6.3	5.8 [5.7–6.0]
Hypertension			+	+	+	+	−	+	+	+	+	+	
Symptoms													
Palpitation			+	+	+	−	−	−	+	−	+	−	
Headache			−	+	−	+	−	+	−	−	−	−	
Weight loss			+	−	+	−	−	−	–	–	–	–	
Constipation			–	–	–	+	–	+	–	–	–	–	
Adrenal tumor			+	+	+	+	+	–	–	–	–	–	
Tumor size	mm		16	42	36	12	27						
CT value of the tumor	HU		− 5	13	45	NA	29						
MIBG uptake intensity													
A/L ratio			1.64	1.35	1.12	1.49	1.09	1.50	1.05	1.46	1.19	2.33	1.29 [1.13–1.67]
Lateral adrenal ratio			1.42	1.47	1.14	1.32	1.07	1.27	1.18	1.01	1.05	1.94	1.22 [1.09–1.39]

### Potential association of FGFR4 variant between AMH and adrenocortical adenomas

**Figure 5:**
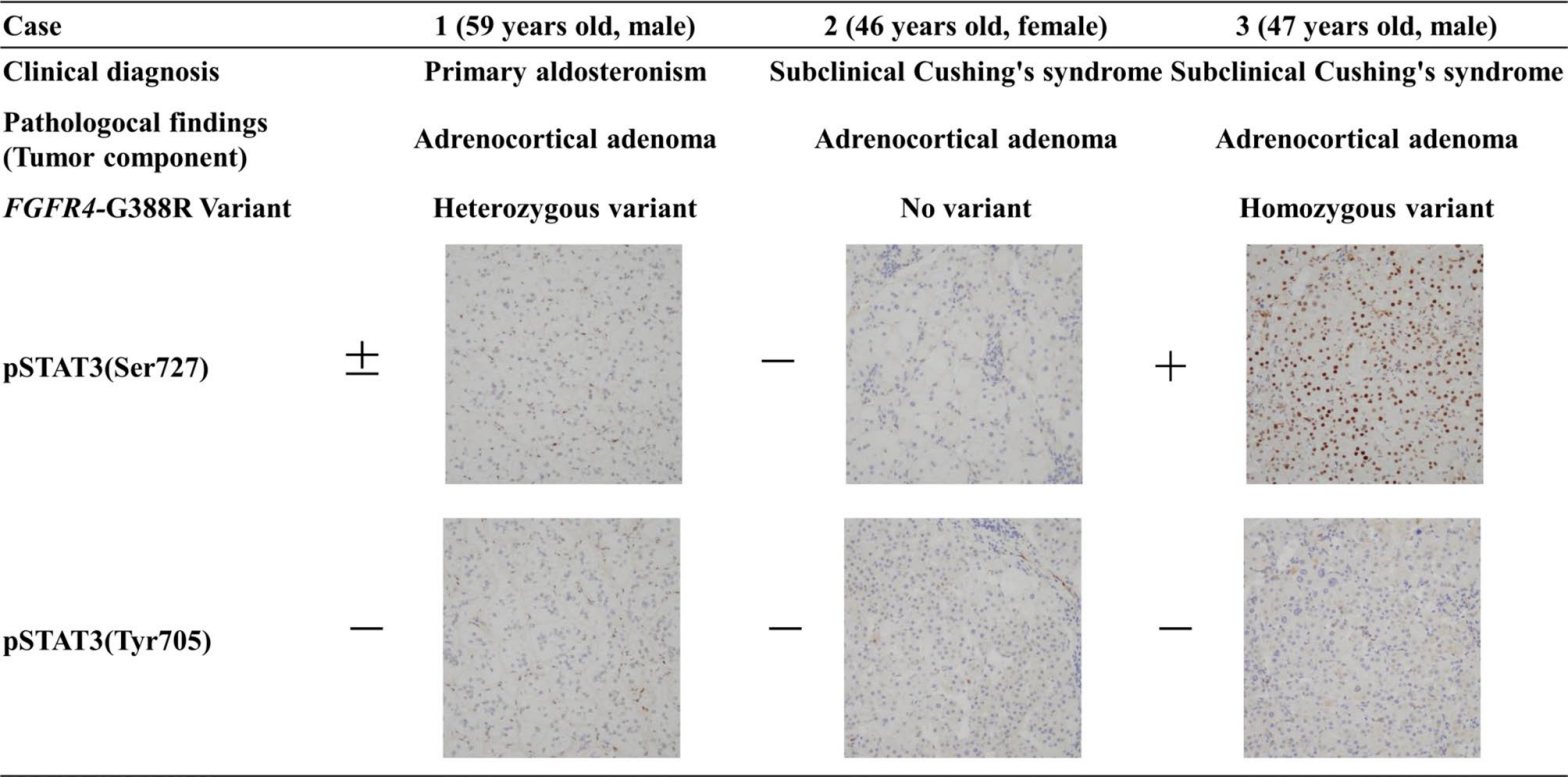
Sequencing analysis in *FGFR4*-G388R variant and immunostaining of phosphorylated STAT3. Phospho-serine (p-S)727 STAT3 was strongly positive in the nucleus of the adrenocortical adenoma component of Case 3, and weakly positive in Case 1. Phospho-tyrosine (p-Y)705 STAT3 was not detected in any case.

Since *FGFR4*-G388R variant has been shown to be a possible initiator of adrenocortical adenoma within pheochromocytoma^19^, we investigated the association between this variant and these adrenocortical adenomas besides AMH rather than mutations in protein kinase A (PKA) pathway, well-known genetic abnormalities in cortisol-producing adenomas^20^. We detected a somatic homozygous *FGFR4-*G388R variant from the adrenocortical adenoma specimens of Case 3, a heterozygous variant from Case 1, and wild-type *FGFR4*- G388R from Case 2. The *FGFR4*-G388R variant is known to promote tumorigenesis through specific serine phosphorylation (p-S) 727 of STAT3^16^. As expectedly, nuclear p-S727 STAT3 positive cells were found in the adrenocortical adenoma components in Cases 1 and 3 (Case 3 > Case 1) in association with *FGFR4* variant status, while no tyrosine phosphorylation of STAT3, a downstream signal of wild-type FGFR4, was found in any tumors. However, the association between AMH and the *FGFR4* variant was not a common change in at least 3 cases. Since this particular change may have potential pathogenic implications, further investigation is needed in this regard (Fig. 5).

## Discussion

This study showed that AMH was present in cases with bilateral adrenal uptake of ^123^I-MIBG scintigraphy with mild CA or MN excess and that the coexisting adrenal tumor may be adrenocortical adenomas. We showed three cases matched with the above conditions and demonstrated their pathological features. Furthermore, retrospective analysis of patients who had undergone ^123^I-MIBG scintigraphy revealed their similar clinical features to CA excess. This result may be meaningful as a suggestion for cases with these diagnostic dilemmas.

There are limited reports on the clinical features of AMH. However, more than half of the AMH cases are related to hereditary diseases, including mainly MEN2, and NF1^21^. Loss of heterozygosity for chromosomal regions associated with pheochromocytoma development in MEN2 has also been observed in AMH. Therefore, it is thought that AMH should be regarded as pheochromocytoma precursor lesions and not as non-neoplastic hyperplasia^13^. According to the WHO classification 2017, PPGL is now recommended to be treated as malignant tumors that may metastasize. However, in AMH that does not meet these diagnostic criteria, follow-up may be interrupted. If AMH is suspected and there is a possibility of subsequent pheochromocytoma, it may be necessary to continue patient follow-up, which should be considered further. In Case 1, urinary MN decreased after surgery (0.31 mg/day to 0.17 mg/day), and the symptom of weight loss associated with excess CA disappeared. In our 3 cases (Cases 1 to 3) in which AMH could be confirmed, no further adrenal tumor appearance or elevated CA or MN levels is observed at present, 1 to 4 years after surgery.

In the evaluation of AMH, it is necessary to rule out atrophy of the adrenal cortex. ACTH could be suppressed in the presence of cortisol autonomous secretion, which can lead to cortical atrophy. In the present cases, ACTH levels in Case 2 and 3 were relatively low means a possibility of cortical atrophy. Serum dehydroepiandrosterone sulfate (DHEA-S) levels, which is also downstream of ACTH was relatively low but within normal range [378 ng/mL (reference range: 190–2310 ng/mL)] in Case 2. Serum DHEA-S levels in Case 3 was also within normal range but relatively high considering his plasma ACTH levels [3129 ng/mL (reference range: 700–4950 ng/mL)]. Therefore, we suspected that plasma ACTH levels in Cases 2 and 3 may not have declined chronically but recently. We carefully observed the thickness of these adrenal cortex, especially in clinically subclinical Cushing’s syndrome cases. We confirmed that there was no atrophy of the adrenal cortex in these 3 cases.

Contrary to the preoperative expectations of pheochromocytomas, all the tumors from the 3 patients who had undergone adrenalectomy were diagnosed with adrenocortical adenomas, which showed positive for CYP11B1 with AMH in the associated adrenal parenchyma. It has been suggested that there may be an interaction between the adrenal medulla and cortex. Although coexistent cases of pheochromocytoma and cortisol-secreting adrenocortical adenoma have been previously reported^22–24^, it is quite rare. Even rarer, an adrenal corticomedullary mixed tumor has been shown, including in our group^19^. In contrast, AMH has been reported to coexist with adrenocortical adenomas with a relatively high prevalence (26%)^21^, suggesting that there is some pathological interaction between cortisol and CA excess, and which may be more frequently associated with AMH than pheochromocytomas. However, few reports of adrenocortical tumors associated with MEN2 or NF1 have been reported. Therefore, we think that a second hit is needed for the mechanism of adrenocortical tumors associated with AMH. Since this study is based on the results of only three cases, further case study is necessary to conclude this pathological hypothesis.

Glucocorticoids are well-known to induce phenylethanolamine *N*-methyltransferase (PNMT), an adrenaline synthetic enzyme^25^, which results in increased CA levels. Meanwhile, CA may also induce pituitary ACTH secretion^26^. Increased cortisol levels in patients with pheochromocytomas but not those with paragangliomas has been reported^11^, suggesting that paracrine, rather than endocrine CA action, plays a more essential role in inducing cortisol secretion^27^. This indicates that there should be cortical and medullary interactions, but their physiological and pathological roles remain unclear.

Regarding with the association between CA excess and adrenocortical adenomas, we have previously reported that the *FGFR4*-G388R variant is involved in the pathogenesis in complication of pheochromocytoma and cortisol-producing adenoma^19^. Therefore, to elucidate the possible mechanism of cortical adenoma formation in AMH, we performed genetic analyses of this gene variant of the tumors from these 3 cases of adrenalectomy. Although the status of *FGFR4*-G388R was strongly associated with the serine phosphorylation of STAT3, a specific downstream activator of this variant^19^ in these 3 tumors, there was no clear consistency between this gene variant and the cortical adenomas associated with AMH.

It is sometimes difficult to determine whether the ^123^I-MIBG accumulation site is in the tumors or in the adrenal gland parenchyma, as in our present cases. From a pathological point of view, we detected AMH in all the resected cases, where it was not located in the adrenal tumor but in the bilateral adrenal parenchyma, suggesting ^123^I-MIBG accumulation in the AMH. In this study, we confirmed the specimens of 34 patients with ^123^I-MIBG accumulation in the unilateral adrenal gland (Fig. 4) showed no AMH, including their associated adrenal parenchyma (data not shown), indicating that AMH may occur as a bilateral pathology. Taken together with the bilateral rate of 10% in pheochromocytoma, it seems likely that AMH is partly involved in this condition.

The limitation of this study is that pathological examination was possible only in 3 of the 10 patients who showed bilateral ^123^I-MIBG accumulation. Therefore, it has not been confirmed whether the remaining 7 cases had AMH, and 2 adrenal tumors were cortical adenomas. In addition, the present study is a cross-sectional study at the time of ^123^I-MIBG scintigraphy, and these cases have not been evaluated longitudinally. It will be necessary to track changes in CA and MN levels in these cases and determine whether new adrenal tumors will appear. Furthermore, the criteria for conducting ^123^I-MIBG in this study are not unified. Therefore, our subjects may include cases that are not difficult to diagnose. We could be analyzed only three adrenalectomy cases. However, the fact that AMH was found in all the three, who underwent surgery, out of the 10 patients identified from the 164 patients who had undergone ^123^I-MIBG scintigraphy was considered to be a significant phenomenon in this rare disease we think. It suggests that this complication is not a coincidence.

In conclusion, the present study showed that adrenocortical adenomas with AMH can be a differential diagnosis in patients with bilateral adrenal uptake of ^123^I-MIBG scintigraphy with mild elevation of CA or MN. Cortical medullary interactions may be involved, but larger studies are needed to clarify its pathophysiology.

## Data Availability

The data that support the findings of this study are available from the corresponding author, [HF: fukuokah@med.kobe-u.ac.jp], upon reasonable request.
