# Author Correction: Bilateral adrenal uptake of ^123^I MIBG scintigraphy with mild catecholamine elevation, the diagnostic dilemma, and its characteristics

**DOI:** 10.1038/s41598-022-17488-2

**Published:** 2022-08-02

**Authors:** Yuiko Inaba, Masaaki Yamamoto, Shin Urai, Masaki Suzuki, Seiji Nishikage, Maki Kanzawa, Yayoi Aoyama, Tomonori Kanda, Katsumi Shigemura, Hironori Bando, Genzo Iguchi, Yasuhiro Nakamura, Masato Fujisawa, Akihisa Imagawa, Hidenori Fukuoka, Wataru Ogawa

**Affiliations:** 1 Division of Diabetes and Endocrinology, Department of Internal Medicine, Kobe University Hospital, 7-5-2 Kusunoki-cho, Chuo-ku, Kobe, 650-0017 Japan; 2 Department of Internal Medicine(I), Osaka Medical and Pharmaceutical University, 2-7 Daigakumachi, Takatsuki, Osaka 569-8686 Japan; 3 Division of Diabetes and Endocrinology, Kobe University Graduate School of Medicine, 7-5-1 Kusunoki-cho, Chuo-ku, Kobe, 650-0017 Japan; 4 Department of Diagnostic Pathology, Kobe University Hospital, 7-5-2 Kusunoki-cho, Chuo-ku, Kobe, 650-0017 Japan; 5 Department of Pathology, Tohoku University Hospital, 1-1 Seiryo-machi, Aoba-ku, Sendai, Miyagi 980-8574 Japan; 6 Department of Radiology, Kobe University Graduate School of Medicine, 7-5-1 Kusunoki-cho, Chuo-ku, Kobe, 650-0017 Japan; 7 Division of Urology, Department of Organ Therapeutics, Faculty of Medicine, Kobe University Graduate School of Medicine, 7-5-1 Kusunoki-cho, Chuo-ku, Kobe, 650-0017 Japan; 8 Department of Public Health, Kobe University Graduate School of Health Science, 7-5-1 Kusunoki-cho, Chuo-ku, Kobe, 650-0017 Japan; 9 Division of Development of Advanced Therapy for Metabolic Disease, Kobe University Graduate School of Medicine, 7-5-1 Kusunoki-cho, Chuo-ku, Kobe, 650-0017 Japan; 10 Medical Center for Student Health, Kobe University, 1-1, Rokkodai-cho, Nada-ku, Kobe, 657-8501 Japan; 11 Department of Biosignal Pathophysiology, Kobe University Graduate School of Medicine, 7-5-1 Kusunoki-cho, Chuo-ku, Kobe, Hyogo 650-0017 Japan; 12 Division of Pathology, Tohoku Medical and Pharmaceutical University, 4-4-1 Komatsushima, Aobaku, Sendai, Miyagi 981-8558 Japan

Correction to: *Scientific Reports* 10.1038/s41598-022-13132-1, published online 03 June 2022

The Funding section in the original version of this Article was incomplete.

“Grant-in-Aid for Scientific Research from the Japanese Ministry of Education, Culture, Sports, Science and Technology 19K09003.”

now reads:

“This study was funded by Grant-in-Aid for Scientific Research from the Japanese Ministry of Education, Culture, Sports, Science and Technology 19K09003 and Medtronic".

The original Article has been corrected.

